# Ifosfamide-Induced Malignancy of Ureter and Bladder

**DOI:** 10.7759/cureus.1594

**Published:** 2017-08-22

**Authors:** Aparna Sannu, Resmi Radha, Anitha Mathews, Rari Padmakumari Mony, Anil Prahladan, Francis V James

**Affiliations:** 1 Department of Radiation Oncology, Regional Cancer Centre Trivandrum; 2 Division of Radiation Oncology, Regional Cancer Centre Trivandrum; 3 Division of Pathology, Regional Cancer Centre Trivandrum; 4 Division of Raiodiagnosis, Regional Cancer Centre Trivandrum

**Keywords:** ifosfamide, urinary bladder neoplasms, neoplasms, second primary/chemically induced

## Abstract

Cyclophosphamide-induced bladder malignancy is a well-known entity mediated by its metabolic product, acrolein. There is a significant association between the incidence of hemorrhagic cystitis during treatment and the later development of malignancies. We report a case of multifocal urothelial carcinoma occurring in a patient treated with ifosfamide 19 years ago. No case report of ifosfamide-induced malignancy could be identified in the literature. A brief review of the literature on the relative risks of ifosfamide therapy, the mechanism of bladder toxicity, and suggestions to minimize the deleterious effects of the drug have been done. Ifosfamide should be used in the lowest possible dose and that patients receiving more than 20 grams of the drug should undergo a routine urinalysis for microscopic hematuria. Prophylactic measures such as high fluid intake, frequent voiding, day time administration of the drug, and concomitant use of mesna may decrease the contact time and the concentrations of toxic metabolites on the bladder urothelium

## Introduction

Ifosfamide is an alkylating agent of the nitrogen mustard type [1]. The drug is inactive in its parent form. Active metabolites form cross-links with deoxyribonucleic acid (DNA) with the resultant inhi­bition of DNA synthesis and function. It is a cell cycle nonspecific (CCNS) antineoplastic agent. Its major toxicity affects the urinary tract, which is manifested by dysuria, increased frequency, and hemorrhagic cystitis, and may progress to bladder fibrosis [2]. Uroprotection with hydration and intravenous (IV) mesna is necessary to prevent this. Chronic bladder fibrosis caused by ifosfamide has been associated with an increased theoretical risk of secondary bladder cancer [1].To the best of our knowledge, no case of a secondary bladder malignancy following ifosfamide treatment has been reported so far.

## Case presentation

A 76-year-old man was diagnosed with soft tissue sarcoma of the left thigh 20 years ago. He was referred to us after a wide excision of the same in August 1997. Histopathology was reported as a high-grade malignant pleomorphic fibrous histiocytoma with osteoid and extra-skeletal osteogenic sarcoma. Metastatic workup was negative. In view of the high-grade nature of the tumor with extensive areas of necrosis and large areas of osteoid, it was decided to treat him with adjuvant chemotherapy, followed by local radiotherapy. He was given adjuvant chemotherapy with four cycles of cisplatin, ifosfamide (1.8 mg/m2), and adriamycin, followed by external beam radiotherapy to the tumor bed (60 Gray in 30 fractions), which was completed in January 1998. He was on regular follow-up for a few years.

**Figure 1:**
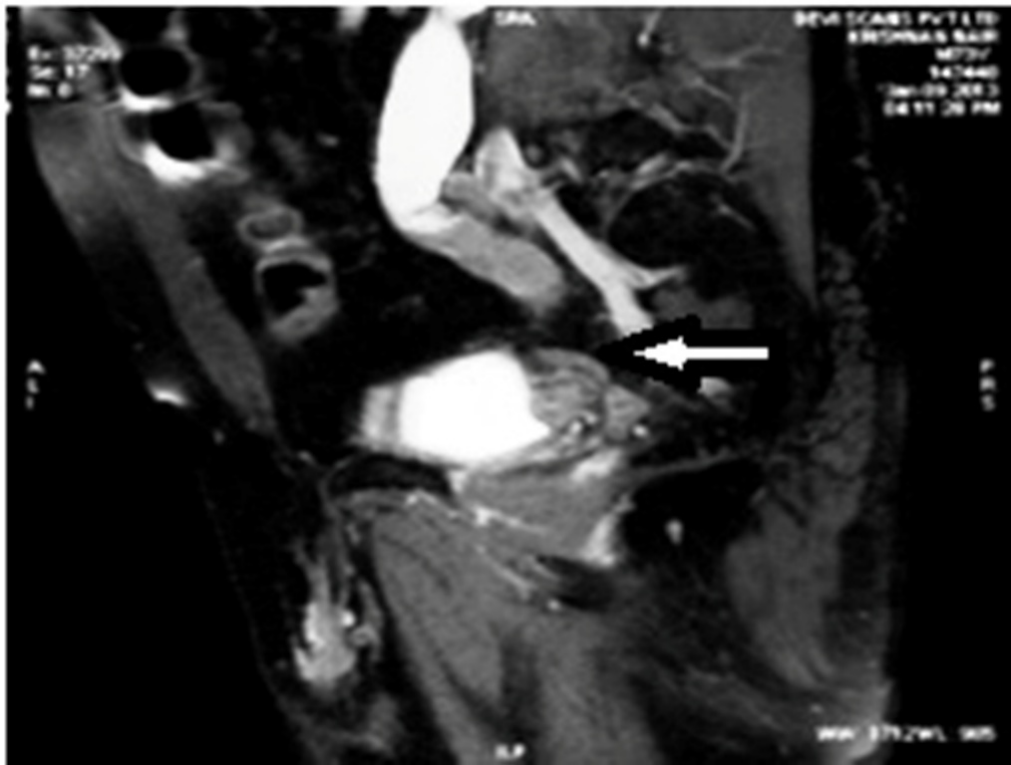
Sagittal T1 weighted post-contrast magnetic resonance image showing an irregular filling defect within the distal ureter with proximal dilatation. The distal ureter appears to be completely occluded by the lesion.

He was evaluated at a local hospital for hematuria in 2014 and was detected to have a carcinoma involving the right ureter (Figure 1).

**Figure 2:**
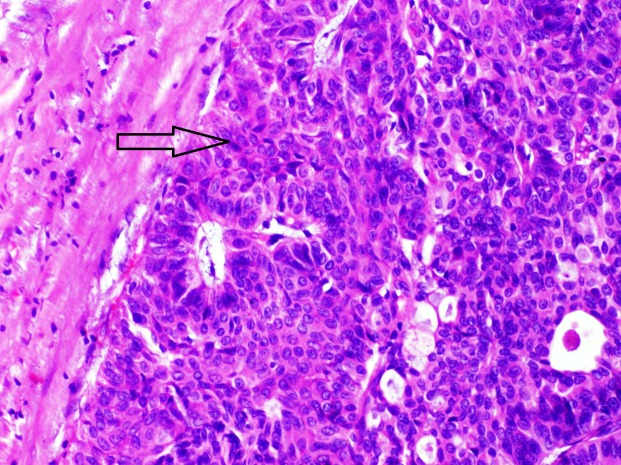
Haematoxylin and eosin staining (H&E) 400x papillary urothelial carcinoma grade II-III in ureterectomy specimen

For this, he underwent a right radical nephroureterectomy in October 2014. Histology revealed transitional cell carcinoma grade II-III, involving the ureter (Figure 2), with pleomorphic changes in the kidney.

**Figure 3:**
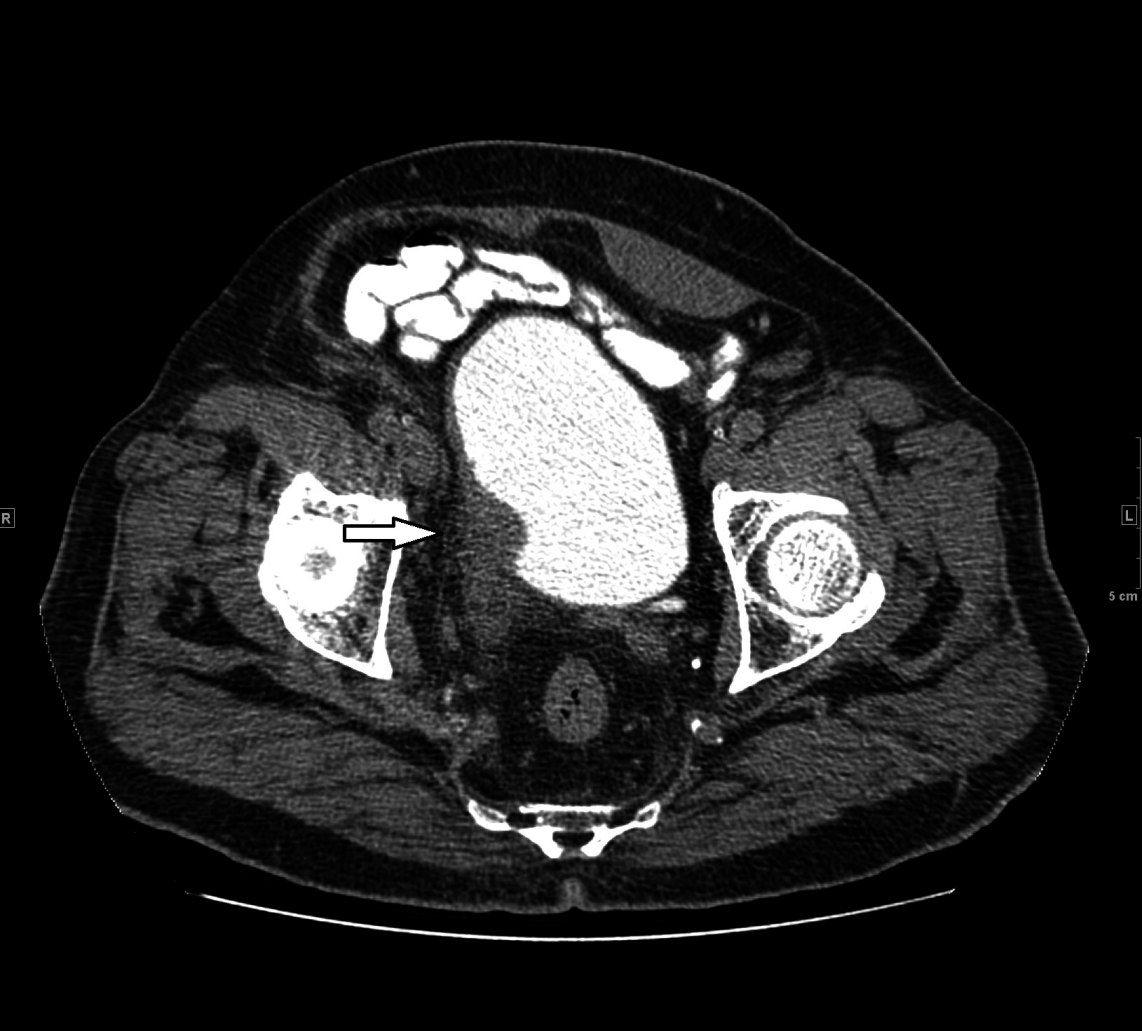
Delayed axial post-contrast computed tomography image showing irregular thickening of the right posterolateral bladder wall involving the ureterovesical junction

Subsequently, he developed a malignant growth in the bladder (Figure 3) for which a transurethral resection of the bladder tumor (TURBT) was done in October 2015.

**Figure 4:**
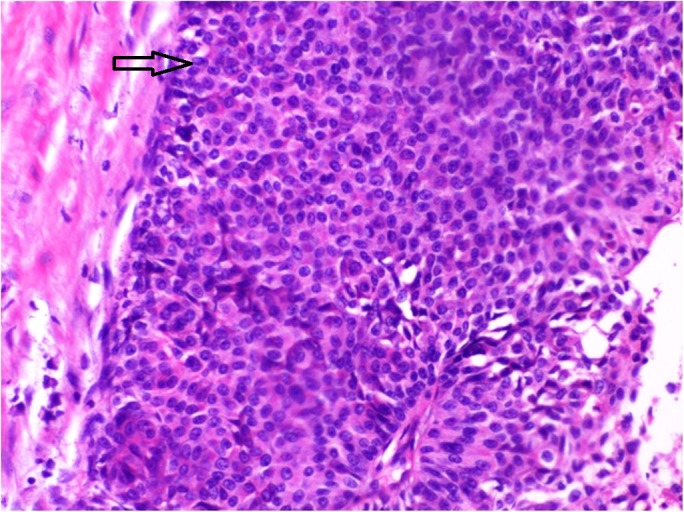
Haematoxylin and eosin staining (H&E) 200x showing papillary urothelial carcinoma grade II in the transurethral resection of the bladder tumor specimen

Histology again showed transitional cell carcinoma (Figure 4).​​​​​​​

Following the surgery, he was kept on follow-up. In February 2016, a computed tomography (CT) scan showed multiple metastases in the pelvic nodes and in the soft tissue deposits in the iliacus muscle. He was referred back to our center for further management. Histology was reviewed by our pathologist and was reported as an infiltrating papillary urothelial carcinoma of the ureter infiltrating the wall, grade II-III in the ureterectomy specimen, and papillary urothelial carcinoma grade II in the TURBT specimen. He is currently undergoing chemotherapy with carboplatin and gemcitabine.

## Discussion

The prolonged survival of cancer patients with improved treatment strategies has made it possible to observe the late effects of treatment modalities, in particular, the occurrence of second primary cancers. The cumulative risk of all second malignancies 20 years after the first diagnosis of ovarian cancer is 18% compared with a population risk of 11.5% [3]. It is estimated that the risk of cancer survivors developing a second primary cancer, increases with the length of survival [4-5]. The first case report on cyclophosphamide-induced transitional cell carcinoma of the bladder for a patient with lymphoma was reported in 1971 [6]. A case control study by Kaldor et al. showed a four-fold increase in the risk for developing bladder cancers for patients treated with cyclophosphamide for primary ovarian malignancy [7]. In a study of 6,000 lymphoma patients treated with cyclophosphamide, the relative risk of developing bladder carcinoma was found to be associated with the cumulative dose of the drug administered [5]. There are several case reports and retrospective studies describing cyclophosphamide use for the treatment of malignancies, e.g., non-Hodgkin lymphoma and autoimmune disorders, such as Wegener's granulomatosis, were responsible for second malignancies involving the urinary tract. Cyclophosphamide is metabolized in the liver to various chlormethine metabolites, which are responsible for the therapeutic effect and acrolein, which mediates the toxic effect to the urothelium-like bladder necrosis, fibrosis, and hemorrhagic cystitis [2]. A significant association between the incidence of hemorrhagic cystitis during treatment and the later development of malignancies has been brought out in some of these studies. Cigarette smoking, exposure to aryl amines (in workers in the organic chemical, dye, rubber, and paint industries), phenacetin abuse, familial history of bladder malignancy, cyclophosphamide therapy, and nonglomerular hematuria are documented risk factors for urothelial cancer.

Intensive chemotherapy has been one of the main treatments for osteosarcoma and because the survival rates of osteosarcoma have greatly increased, large-scale studies have documented an increased incidence of second malignancies in these patients [8-9].

In a retrospective study by Kim et al., a high cumulative alkylating agent dose was found to be an independent risk factor for second malignant neoplasms and its early development after osteosarcoma treatment [10]. In our patient, the cumulative dose of ifosfamide used was 21.6 gm/m2.

Ifosfamide is a prodrug that requires metabolic activation by hepatic cytochrome P450 isoenzymes to yield the cytotoxic and urotoxic compound acrolein and alkylating isophosphoramide mustard as well as multiple other nontoxic products. The exact mechanism of the action of ifosfamide has not been determined, but its cytotoxic action is primarily through DNA crosslinks caused by alkylation through isophosphoramide mustard at the guanine N-7 positions. The formation of inter- and intra-strand cross-links in the DNA results in cell death.

Nonclinical toxicology studies have documented the potential for carcinogenesis and mutagenicity [1].

Ifosfamide has been shown to be carcinogenic in rats when administered by intraperitoneal injection at about 3% of the daily human dose on a mg/m2 basis. Female rats had a significantly higher incidence of uterine leiomyosarcomas and mammary fibroadenomas than controls.

The mutagenic potential of ifosfamide has been documented in bacterial systems in vitro and mammalian cells in vivo. In vivo, ifosfamide has induced mutagenic effects in mice and Drosophila melanogaster germ cells and has induced a significant increase in dominant lethal mutations in male mice as well as recessive sex-linked lethal mutations in Drosophila.

Treatment with ifosfamide involves the risk of secondary tumors and their precursors as late sequelae [1]. The risk of myelodysplastic alterations, some progressing to acute leukemias, is increased [1]. Other malignancies reported after the use of ifosfamide or regimens with ifosfamide include lymphoma, thyroid cancer, and sarcomas [1]. The secondary malignancy may develop several years after chemotherapy has been discontinued.

Prophylactic measures, such as high fluid intake, frequent voiding, the daytime administration of the drug, and the concomitant use of mesna may decrease the contact time and concentrations of toxic metabolites on the bladder urothelium.

## Conclusions

In our case, the patient was treated with ifosfamide. The fact that the reported patient had two subsequent malignancies in well-separated regions of the urinary tract suggests the possibility of a field cancerization effect induced by ifosfamide. To the best of our knowledge, this is the first case report linking ifosfamide and the development of urinary tract malignancy.
